# Two-Year Follow-up of a Group-Sequential, Multicenter Randomized Controlled Trial of a Subacromial Balloon Spacer for Irreparable Rotator Cuff Tears of the Shoulder (START:REACTS)

**DOI:** 10.1177/03635465251326891

**Published:** 2025-03-28

**Authors:** Aminul Haque, Helen Parsons, Nick Parsons, James Mason, Iftekhar Khan, Nigel Stallard, Martin Underwood, Charles Hutchinson, Tom Lawrence, Steve Drew, Rebecca Kearney, Andrew Metcalfe

**Affiliations:** * Warwick Clinical Trials Unit, Medical School, University of Warwick, Coventry, UK; ‡ University Hospitals Coventry and Warwickshire NHS Trust, Coventry, UK; § Bristol Medical School, University of Bristol, Bristol, UK; Investigation performed at Warwick Clinical Trials Unit, Medical School, University of Warwick, Coventry, UK

**Keywords:** shoulder, rotator cuff, subacromial space, randomized control trial

## Abstract

**Background:**

The best management of irreparable rotator cuff tears remains uncertain, with multiple new techniques introduced over the past 2 decades. Two options for treatment are arthroscopic debridement and biceps tenotomy, or the subacromial balloon spacer. Early trial results favored the former option, but the 2-year results have not yet been reported.

**Purpose:**

To report the 2-year follow-up outcomes of the START:REACTS trial, investigating the use of a subacromial balloon spacer for irreparable rotator cuff tears of the shoulder.

**Study Design:**

Randomized controlled trial; Level of evidence, 1.

**Methods:**

Eligible participants had an irreparable rotator cuff tear, intrusive symptoms requiring surgery, and previous unsuccessful nonoperative care. Participants were randomized 1:1 to debridement of the subacromial space with biceps tenotomy (debridement only) or the same procedure with the addition of the subacromial balloon spacer (debridement with device). The 12-month primary outcome was previously reported; this article presents the 24-month results. Linear regression models were used to analyze the 24-month data.

**Results:**

Recruitment stopped early at the preplanned interim analysis, with 117 participants in the trial. A total of 99 (85%) participants out of 117 were followed up to 24 months. At 24 months, a significant difference in the Oxford Shoulder Score was not found (95% CI, –7.9 to 0.4; *P* = .08). The Western Ontario Rotator Cuff score (mean difference, –10.1; 95% CI, –19.5 to −0.8; *P* = .04) and Patient Global Impression of Change (odds ratio, 0.4; 95% CI, 0.2 to 0.8; *P* = .015) were found to significantly favor debridement only. The EQ-5D-5L (mean difference, −0.009; 95% CI, −0.107 to 0.088; *P* = .85) and satisfaction scores (odds ratio, 0.6; 95% CI, 0.3 to −1.2; *P* = .14) were not significantly different. Complications were evenly matched between groups over 24 months.

**Conclusion:**

Participants continued to show better results in the debridement-only group compared with the group who had debridement with the InSpace balloon. Therefore, we do not recommend the subacromial balloon spacer for the treatment of irreparable rotator cuff tears.

Rotator cuff tears are a common clinical presentation,^20^ and although many tears are asymptomatic, a high proportion of people with a rotator cuff tear will go on to develop symptoms including pain and reduced function.^21,23,29,33^ Many people improve after nonoperative care including physical therapy, but some are treated surgically.^13^ Although rotator cuff repair is a well-accepted surgical treatment for tears, including massive cuff tears, some tears cannot be repaired.^8^ The treatment of irreparable tears is controversial, with numerous techniques proposed and few randomized trial data to determine which is most effective.^6,7,26,30,34,27^ Arthroscopic debridement, often including biceps tenotomy if the tendon is intact, is a common treatment, and case series suggest patient benefit. However, insufficient evidence from clinical trials is available, and it remains unknown whether arthroscopic debridement provides adequate benefit or whether other treatment options would be better.^14,15,25^

In 2013, the InSpace subacromial balloon spacer device (Stryker, USA) was introduced into UK orthopaedic practice as a potential treatment option. It is a saline-filled balloon made of biodegradable (dissolvable) synthetic material and is inserted in the subacromial space, after an arthroscopic debridement has been performed. It cushions the humerus from pressing on the bone above it, the acromion, when the deltoid is active and during abduction of the arm, potentially improving the biomechanics of the shoulder and reducing pain.^28^ In May 2016, an interventional procedure guidance document was published by the National Institute for Health and Care Excellence (NICE) demonstrating very limited evidence for use of the InSpace device.^22^ A research recommendation was made by NICE to assess its effectiveness.

We conducted a group-sequential, double-blind, multicenter, randomized controlled trial of a subacromial balloon spacer device for irreparable rotator cuff tears of the shoulder (START:REACTS) to investigate the efficacy of using the balloon device for people needing operative intervention for irreparable rotator cuff tears. In 2022, the START:REACTS primary results were published showing that participants allocated to debridement alone (control) had better outcomes at 12 months and that women had worse results with the balloon compared with men in prespecified subgroup analysis.^18^ We present the 24-month follow-up data here. We also conducted an exploratory analysis to investigate whether the observed differences between men and women are due to differences in shoulder size or baseline strength.

## Methods

### Trial Recruitment and Study Population

The study design is described in detail elsewhere.^16-18,24^ In brief, the START:REACTS trial was a 1:1 parallel-designed, group-sequential, and pragmatic multicenter randomized controlled trial in the United Kingdom. The trial compared arthroscopic debridement and biceps tenotomy versus arthroscopic debridement and biceps tenotomy with the balloon device. This ensured that the study had a comparator that was an accepted and widely used surgical treatment but also ensured a robust experiment where the only difference between the 2 interventions was the balloon device. Participants and investigators were blinded to the interventions, although participants were informed of their allocation after publication of the 12-month primary outcome, in agreement with the ethics committee. As a result, 4 participants included in this analysis were unblinded for their 24-month outcome assessment. The trial was undertaken using a novel adaptive design framework, which we referred to as a randomized, efficient, adaptive design clinical trial in surgery (REACTS).^24^ The design of the study allowed early stopping of the trial while maintaining the validity and integrity of the trial. The study was approved by the Coventry and Warwickshire research ethics committee (study registration No. ISRCTN17825590).

The trial recruited participants between June 1, 2018, and July 30, 2020, and they were followed up for 24 months. Potential participants were screened at shoulder clinics and surgery waiting lists. To be eligible for the trial, participants needed to have a rotator cuff tear that was determined to be irreparable (ie, it could not technically be repaired) by the treating clinician, have intrusive symptoms that warranted surgery, and have undergone nonoperative care that was unsuccessful. Participants were not eligible if they were found to meet any of the following criteria: were younger than 18 years; had advanced glenohumeral osteoarthritis, subscapularis deficiency, pseudoparalysis, symptomatic ipsilateral shoulder disorder, a proximal humerus fracture, or a neurological or muscular condition that would affect shoulder function; needed interposition or tendon transfer; were unable to complete trial procedures; or were unfit for surgery. Further details of these criteria can be found in the study protocol.^17^ Eligibility was confirmed at initial arthroscopy at the time of surgery, and participants were excluded intraoperatively if their rotator cuff was found to be repairable, they had advanced glenohumeral osteoarthritis, or the subscapularis was found to be deficient.

### Interventions and Randomization

Participants were randomized intraoperatively after the initial arthroscopic assessment and underwent debridement of the subacromial space, with minimal bony debridement and retention of the coracoacromial ligament with biceps tenotomy (if biceps was intact) following an agreed-upon surgical protocol and technique video.^19^ Biceps tenotomy was recommended if the biceps was found to be intact; it was documented intact in 38 of 61 patients in the debridement-only group and 39 of 56 patients in the debridement-with-device group.

If found eligible, participants were allocated in a 1:1 ratio to either debridement only or debridement with device, the latter of which entailed insertion of the InSpace balloon device. Randomization was performed by minimization with a random element, stratifying by study site, age group (≥70 or <70 years), sex, and intraoperatively measured tear size (≥3 or <3 cm). The randomisation system was accessed via a web-based portal or through an automated telephone system, although a staffed back-up system was also available. For participants who were allocated to the balloon procedure, the surgical technique followed the manufacturer’s recommendation for sizing, insertion, and deployment of the balloon. After surgery, both groups received postoperative information and a standardized physical therapy program that was developed by an expert panel during the set-up phase of the trial.^11^

### Outcomes

The primary outcome measure was the Oxford Shoulder Score (OSS) at 12 months. In the original study design, the primary outcome was the Constant score; however, due to the coronavirus pandemic, the primary outcome was changed to the OSS. Because the Constant score requires patients to meet face-to-face at hospitals, this would expose patients to unnecessary risk at the time of the pandemic. The decision to change the primary outcome was agreed by the independent oversight committees. The OSS is a validated scoring system used to assess the degree of pain and disability caused by shoulder pathology.^4,5^ The score ranges from 0 to 48, where higher scores correspond to better functional outcomes.

Secondary outcomes included the OSS at other time points, the Western Ontario Rotator Cuff Index (WORC),^9,12^ EQ-5D-5L,^1,10^ satisfaction, and the Participant Global Impression of Change scale. The WORC is a rotator cuff–specific instrument evaluating the change in symptoms and functional ability related to the shoulder. It measures 5 domains: physical symptoms, sports/recreation, work, lifestyle, and emotions. We used an adjusted score, which reverses the original scale of 0 to 2100, resulting in a final score that ranges from 0 to 100, with higher scores corresponding to better outcomes. The EQ-5D-5L^1,10^ is a validated outcome used to assess generic health-related quality of life, covering 5 domains: pain, mobility, anxiety, self-care, and daily activities. The UK 3L crosswalk health scores of the EQ-5D-5L were used, which range from −0.594 to 1, with 1 representing perfect health and 0 being equivalent to death.^31^ Treatment satisfaction was collected using a 5-point scale, and the Participant Global Impression of Change score was also used, which is a 7-point discrete scale that assesses the participant’s perception of improvement.

### Sample Size

The study had statistical power to detect a target difference of 6 points in the OSS. A maximum target sample size of 221 was established in the design phase of the study to achieve 90% power, assuming 85% follow-up. Following the preplanned adaptive design that was used for the trial, the study was stopped early at the first interim analysis with 117 participants randomized.^17,24^ The results of that interim analysis were that the test statistics had exceeded the prespecified statistical boundaries for futility, thus recommending early termination of the study (for a detailed summary of the decision process, see the appendix to the 12-month results paper).^18^ The patients who had already been recruited were then followed up for the remainder of the trial.

### Statistical Analyses

The 24-month analyses were conducted on an intention-to-treat basis, following the procedures used to assess the 12-month data. A fixed-effects linear regression model was used to analyze the OSS. The model was adjusted for the baseline score and stratification variables (age group, sex, and tear size). The recruiting site was explored in the random-effects model but not reported due to poor fitting. Similarly, this method was used to analyze the WORC and the EQ-5D-5L.

Prespecified subgroup analyses to investigate the effects of the allocation groups on participant age, sex, and tear size were planned, which were modeled using the above approach with an additional interaction term. An ordinal logistic regression model was used to analyze the outcomes for patient satisfaction and global impression of change. Finally, post hoc analyses were conducted on participant height and baseline strength. Regression models were used to make an assessment using the same methods as the primary analyses. We hypothesized that the sex differences may be due to how the InSpace balloon fits into the shoulder. We used participants’ height as a proxy measure of shoulder size and added this into the sex-subgroup model previously used. Baseline strength, as measured for the baseline Constant score (following a standardized protocol and using a dynamometer),^2^ was analyzed to investigate the relationship to patient outcomes at 12 and 24 months. An additional sensitivity analysis was conducted on patients without torn subscapularis. These analyses were undertaken using adjusted linear regression models as outlined for the primary analyses. EQ-5D-5L was interpreted at individual timepoints and used to generate quality-adjusted life years (QALYs) across the 24-month time horizon. Mechanisms of missingness of data were explored and multiple imputation methods applied to impute missing data. Using the trapezoidal rule, we calculated the area under the curve of health status scores, providing patient-level QALY estimates.

## Results

At 24 months, we obtained data from 99 participants, 85% of the total 117 randomized, with 17 (15%) lost to follow-up and 1 death (unrelated to the study interventions). The number of participants returning data for each allocation group was 45 in the debridement-with-device group and 54 in the debridement-only group. Descriptive statistics of those who remained in the study and those who were lost to follow-up are shown in Table 1 and in the consort diagram in Appendix Figure A1 (available in the online version of this article).

**Table 1:** Baseline Characteristics of 24-Month Participants^*a*^

Baseline Characteristics	24-Month Questionnaire Received (n = 99)	Lost to Follow-up (n= 18)	All Randomized Participants (n = 117)
n (% of randomized)	99 (85)	18 (15)	117
Allocation group			
Debridement only	54 (55)	7 (39)	61 (52)
Debridement with device	45 (45)	11 (61)	56 (48)
Age, y	67 ± 8	67 ± 8	67 ± 8
<70 y	55 (55)	14 (78)	69 (59)
≥70 y	44 (44)	4 (22)	48 (41)
Sex			
Male	53 (53)	14 (78)	67 (57)
Female	46 (47)	4 (22)	50 (43)
Rotator cuff tear size			
Large	93 (94)	18 (100)	111 (95)
Medium or small	6 (6)	00 (0)	6 (5)
Body mass index, kg/m^2^	30.4 ± 5.4	30.5 ± 5.2	30.5 ± 5
Height, cm	167 ± 9	168 ± 10	168 ± 10
Weight, kg	86 ± 19	87 ± 19	87 ± 19
Side of shoulder affected			
Left	32 (32)	6 (33)	38 (33)
Right	67 (68)	12 (67)	79 (68)
Participants left or right-handed			
Left	13 (13)	2 (11)	15 (13)
Right	85 (86)	16 (89)	101 (86)
Missing	1 (1)	00 (0)	1 (1)
Dominant or nondominant shoulder affected			
Dominant	64 (65)	10 (56)	74 (63)
Nondominant	34 (34)	8 (44)	42 (36)
Missing	1 (1)	0	1 (1)
Symptom duration, y	4.6 ± 6	6.6 ± 9.7	4.9 ± 7
Participants with previous trauma or injury that may have caused tear			
Yes	66 (67)	14 (78)	80 (68)
Missing	1 (1)	00 (0)	1 (0.9)
Other medical conditions			
Yes	83 (84)	15 (84)	98 (84)
Missing	1 (1)	00 (0)	1 (0.9)
Participant smokes			
Yes	7 (7)	2 (11)	9 (8)
Mean, cigarettes per day	10.6 ± 6	8.5 ± 9.2	10 ± 6
Missing	1 (1)	00 (0)	1 (1)
Participant has diabetes			
Yes	16 (16)	2 (11)	18 (15)
Missing	3 (3)	00 (0)	3 (3)
Type 1	00 (0)	00 (0)	00 (0)
Type 2	16 (16)	2 (11)	18 (15)
Treatment method for diabetes			
Insulin	1 (1)	00 (0)	1 (1)
Medication	11 (11)	00 (0)	11 (9)
Diet	5 (5)	02 (11)	7 (6)
Baseline OSS for all participants	22.4 ± 9.0	22.4 ± 8.9	22.4 ± 9.0

a Data are expressed as n (%) or mean ± SD. Percentages are of follow-up status. OSS, Oxford Shoulder Score.

**Figure 1.**
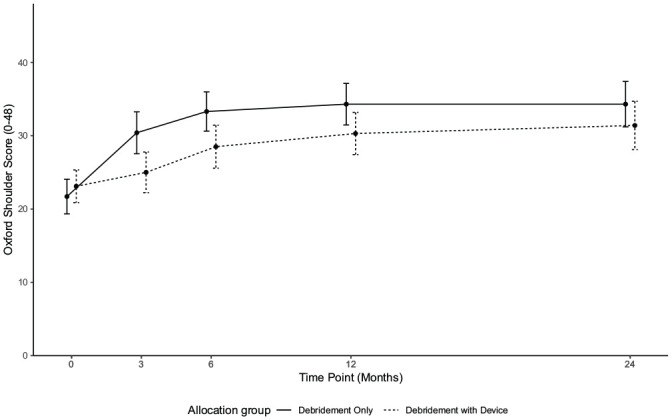
Oxford Shoulder Score means and 95% CI.

In the analyses for the 24-month follow-up, the OSS showed no statistically significant difference between the interventions (adjusted mean difference, –3.8; 95% CI, –7.9 to 0.4; *P* = .075), trending in the direction of debridement only. As shown in Figure 1, the score trajectories of both groups appeared stable between 12 and 24 months, with the mean score of the debridement-with-device group increasing by only 1 point, and the mean score of the debridement-only group remaining static (Table 2). Only 9% of participants at 24 months scored the maximum value, suggesting that the conclusions are unlikely to be affected by a ceiling effect (conventionally defined as >15% of scores at the maximum value). A more detailed density plot of OSS at 12 and 24 months is provided in Appendix Figures A2 and A3 (available online).

**Table 2:** Results for Outcome Measures^*a*^

	Debridement Only (n = 61)		Debridement With Device (n = 56)		Between-Group Difference (95% CI)		
	n (%)	Mean ± SD	n (%)	Mean ± SD	Unadjusted^*b*^	Adjusted^*c*^	*P*
OSS							
12 months	59 (97)	34.3 ± 11.1	55 (98)	30.3 ± 10.9	–4.2^*d*^	–4·2 (–7.8 to −0.6)	.026
24 months	53 (87)	34.3 ± 11.5	45 (80)	31.4 ± 11.3	–2.9	–3.8 (–7.9 to 0.4)	.075
WORC							
12 months	56 (92)	61.6 ± 25.7	51 (91)	51.7 ± 23.5	–9.9	–8.4 (–16.7 to −0.1)	.055
24 months	51 (83)	62.7 ± 25.3	43 (77)	51.7 ± 25.2	–11	–10.1 (–19.5 to −0.8)	.041
EQ-5D-5L							
12 months	58 (95)	0.667 ± 0.287	55 (98)	0.590 ± 0.286	–0.077	–0.015 (–0.08 to 0.12)	.765
24 months	54 (89)	0.638 ± 0.298	44 (79)	0.620 ± 0.245	0.002	–0.009 (–0.107 to 0.088)	.852

a Score ranges: OSS (0-48), WORC (0-100), EQ-5D-5L (–0.594 to 1). Positive values favor debridement with device. OSS, Oxford Shoulder Score; WORC, Western Ontario Rotator Cuff Index.

b Mean difference *t* test.

c Adjusted for baseline score, age group, sex, and tear size.

d REACTS model result (unadjusted).

A statistically significant difference between the intervention groups was observed for the WORC score at 24 months. Participants in the debridement-with-device group had significantly worse shoulder disability (mean adjusted score, –10.1; 95% CI, –19.5 to–0.8; *P* = .040). No statistically significant difference was observed in EQ-5D-5L at 24 months (mean difference, 0.009; 95% CI, −0.107 to 0.088; *P* = .852) or between QALYs over the 24-month period: QALY of 0.591 for debridement only and 0.546 for debridement with device, a difference of 0.045 (*P* = .36). No significant difference was found in the 5-point satisfaction scale between the intervention groups (ordinal regression odds ratio [OR], 0.6; 95% CI, 0.3 to 1.2; *P* = .136 at 24 months). However, a statistically significant difference was found in the Patient Global Impression of Change scale at 24 months (OR, 0.4; 95% CI, 0.2 to 0.8; *P* = .015) (Table 3). The odds of having better outcomes (ie, considerable, better, or slight but noticeable improvement) was lower by 60% for the debridement-with-device group compared with debridement only.

**Table 3:** Results for Satisfaction and Participant Global Impression of Change^*a*^

Transition Scores at 24 Months	Debridement Only (n = 61)	Debridement With Device (n = 56)	Odds Ratio^*b*^ (95% CI)
Overall change since operation (satisfaction)			
Substantially better	27 (48)	17 (30)	0.6 (0.3-1.2) *P* = .136
Moderately better	11 (20)	12 (21)	
No difference	8 (14)	4 (7)	
Moderately worse	4 (7)	7 (12)	
Substantially worse	2 (4)	5 (9)	
Missing	9 (16)	11 (20)	
Change in activity limitations, symptoms, emotions, and overall quality of life since operation			
No change or worse	7 (12)	17 (30)	0.4 (0.2-0.8) *P* = .015
Almost the same	8 (14)	2 (4)	
A little better, no noticeable change	4 (7)	2 (4)	
Somewhat better, change has not made a difference	4 (7)	7 (12)	
Moderately better, slight but noticeable change	1 (2)	5 (9)	
Better, definite improvement with a difference	17 (30)	7 (12)	
Considerable improvement making a huge difference	11 (20)	5 (9)	
Missing	9 (16)	11 (20)	

a Data are reported as counts and percentage of total in intervention group.

b Odds ratio (OR) calculated via adjusted proportional ordered regression. OR <1 favors debridement-only group.

Although a significant difference was found between the intervention groups for female and male outcomes at 12 months,^18^ the interaction effect was not statistically significant at 24 months (–7.8-point difference for women treated with debridement with device; 95% CI, –15.9 to 0.4; *P* = .06). Appendix Table A2 (available online) shows the model results. We found that increasing participant height was not significantly associated with function (OSS) at 12 months (regression coefficient, 0.04; 95% CI, −0.29 to 0.38; *P* = .781) and also was not significant at 24 months (0.05; 95% CI, −0.33 to 0.44; *P* = .789). Baseline abduction strength had no significant association with OSS at 12 months (regression coefficient, −0.3; 95% CI, −0.8 to 0.1; *P* = .150) or 24 months (–0.1; 95% CI, −0.6 to 0.4; *P* = .661). A sensitivity analysis was conducted excluding patients with a torn subscapularis, and the results showed no differences to the effect of the intervention (–3.9-point difference; 95% CI, –9.0 to 1.0).

Safety events and complications were uncommon in either group during the follow-up from 12 to 24 months. Only 2 participants reported further shoulder surgery over the 24-month period. Both had a reverse shoulder arthroplasty, 1 participant in each allocation group. The safety events overall for the trial were balanced between groups (Appendix Table A1, available online).

## Discussion

At 24 months after surgery, no benefit was found for debridement with use of the InSpace device compared with the same treatment without the device. In many of the secondary outcomes, even at 2 years, worse outcomes were observed for the group treated with the device, such as the WORC and the Participant Global Impression of Change. Although a statistically significant difference between allocation groups was not seen in the OSS at 24 months, the magnitude of the group differences changed little between 12 and 24 months.

The precise reason for the differences between the treatment groups is not known and warrants further investigation. We found no evidence of a ceiling effect in scores, and the results were consistent across the different secondary outcomes, so we can be confident that this was not a chance finding. The difference between groups was especially pronounced in women in our primary 12-month analysis.^18^ Our results show a clinically meaningful difference between the 2 groups at 24 months, but this was not statistically significant. As an underpowered exploratory analysis, this may remain a chance finding, but given the size of the difference and the fact that it was statistically significant at 12 months, it is worth considering.

To further explore the reasons for the differences between men and women, we examined the possibility that either shoulder size (using height as a proxy measurement) or baseline strength (taken as part of the Constant score) was related to shoulder function at 12 months, but we found that neither factor was related to our outcomes. Given the large differences in function between sexes observed in the trial, we hypothesized that if shoulder size or baseline strength did influence the response to the InSpace device, then a large effect would also be observed in these analyses. A nonmechanical cause, such as a reaction to the material or other cause of pain or dysfunction, is a possibility but requires further study.

Shortly after the publication of our 12-month results, the findings of a commercially funded noninferiority trial of the InSpace balloon device were published by Verma et al.^32^ They compared the use of the InSpace device to a partial repair of the rotator cuff, a different procedure to our comparator, which has had mixed results reported previously.^3,28^ Although there were some differences in eligibility criteria between Verma’s study and START:REACTS,^17^ the mean ages and the distribution of women and men were similar, as was the reported baseline strength. We were unable to compare baseline range of motion because we used the Constant score definition of pain-free range of motion whereas Verma and colleagues appeared to report full active range of motion (although their measurement protocol has not been published). Verma and colleagues found that the InSpace device was noninferior to partial cuff repair. Given our findings in the current study that baseline strength or the status of subscapularis was not associated with shoulder function or outcomes, it is unlikely that any baseline differences between the populations had a major influence on the results. Hence, we hypothesize that differences between the studies are more likely to be related to the different study designs and comparators.

Extensive debate surrounds the best treatment for patients with irreparable rotator cuff tears, with a multitude of treatment options including debridement, superior capsular reconstruction, partial repair, tendon transfers, and reverse shoulder replacement.^6,7,26,30,34^ The current study has demonstrated that we need high-quality randomized trial evidence to determine which treatments give the best results for our patients, and surgeons should demand to see trial evidence for new devices and techniques. Our study was designed to stop early under appropriate circumstances using a predetermined statistical technique described in detail previously.^24^ Power was retained at little statistical cost by using all of the available data in the study to strengthen the statistical decision rules. The study had sufficient data to stop the trial at 117 participants, which was far fewer than the original maximum sample size, and concluded that the primary outcome at 12 months (OSS) was statistically significant in favor of the debridement-only group. The pattern of outcomes at the 24-month analyses was consistent with the 12-month findings. Using this method allowed us to complete the study at least a year earlier than we might have otherwise, providing important early data to the clinical community. This finding demonstrates the success of the chosen novel statistical approach and shows that adaptive designs can be beneficial to clinical practice, such as reducing risk to patients for ineffective treatments. This was a valuable learning experience and will provide a useful efficient framework for future trials.

### Limitations

Follow-up rates were lower at 24 months compared with 12 months, but an 85% completion rate is consistent with the original power calculation for the primary outcome. It is highly unlikely that any additional data would have changed our core findings, given the strength of the results. Due to the pandemic we did not have any radiological follow-up, which might have given us a better understanding of the observed differences in the patient outcomes. Although the balloon has mostly been used without repair, it has been described for use in combination with partial or full rotator cuff repair, and more studies are needed in those settings. Given that the results with the balloon were worse in our study, such evaluation should be performed in the setting of a well-controlled and monitored randomized trial.^16^

## Conclusion

We performed a blinded randomized multicenter trial comparing arthroscopic debridement with the InSpace device versus arthroscopic debridement alone. Outcomes for those who underwent operation with the device were worse at both 12 and 24 months compared with the same operation but without the device. Due to the additional costs of the device and its lack of benefit, healthcare funds would be better allocated elsewhere. We do not recommend the InSpace device for patients who have an irreparable tear of the rotator cuff.

## Supplemental Material

sj-pdf-1-ajs-10.1177_03635465251326891 – Supplemental material for Two-Year Follow-up of a Group-Sequential, Multicenter Randomized Controlled Trial of a Subacromial Balloon Spacer for Irreparable Rotator Cuff Tears of the Shoulder (START:REACTS) Supplemental material, sj-pdf-1-ajs-10.1177_03635465251326891 for Two-Year Follow-up of a Group-Sequential, Multicenter Randomized Controlled Trial of a Subacromial Balloon Spacer for Irreparable Rotator Cuff Tears of the Shoulder (START:REACTS) by Aminul Haque, Helen Parsons, Nick Parsons, James Mason, Iftekhar Khan, Nigel Stallard, Martin Underwood, Charles Hutchinson, Tom Lawrence, Steve Drew, Rebecca Kearney and Andrew Metcalfe in The American Journal of Sports Medicine
