# Supplementary material for: Two-Year Follow-up of a Group-Sequential, Multicenter Randomized Controlled Trial of a Subacromial Balloon Spacer for Irreparable Rotator Cuff Tears of the Shoulder (START:REACTS)
Source: Am J Sports Med. 2025 Mar 28;53(6):1291–8. doi: 10.1177/03635465251326891 (PMC12044206; doi:10.1177/03635465251326891)
Supplement: sj-pdf-1-ajs-10.1177_03635465251326891 – Supplemental material for Two-Year Follow-up of a Group-Sequential, Multicenter Randomized Controlled Trial of a Subacromial Balloon Spacer for Irreparable Rotator Cuff Tears of the Shoulder (START:REACTS) [file sj-pdf-1-ajs-10.1177_03635465251326891.pdf]

Appendix

Figure A1 CONSORT Diagram

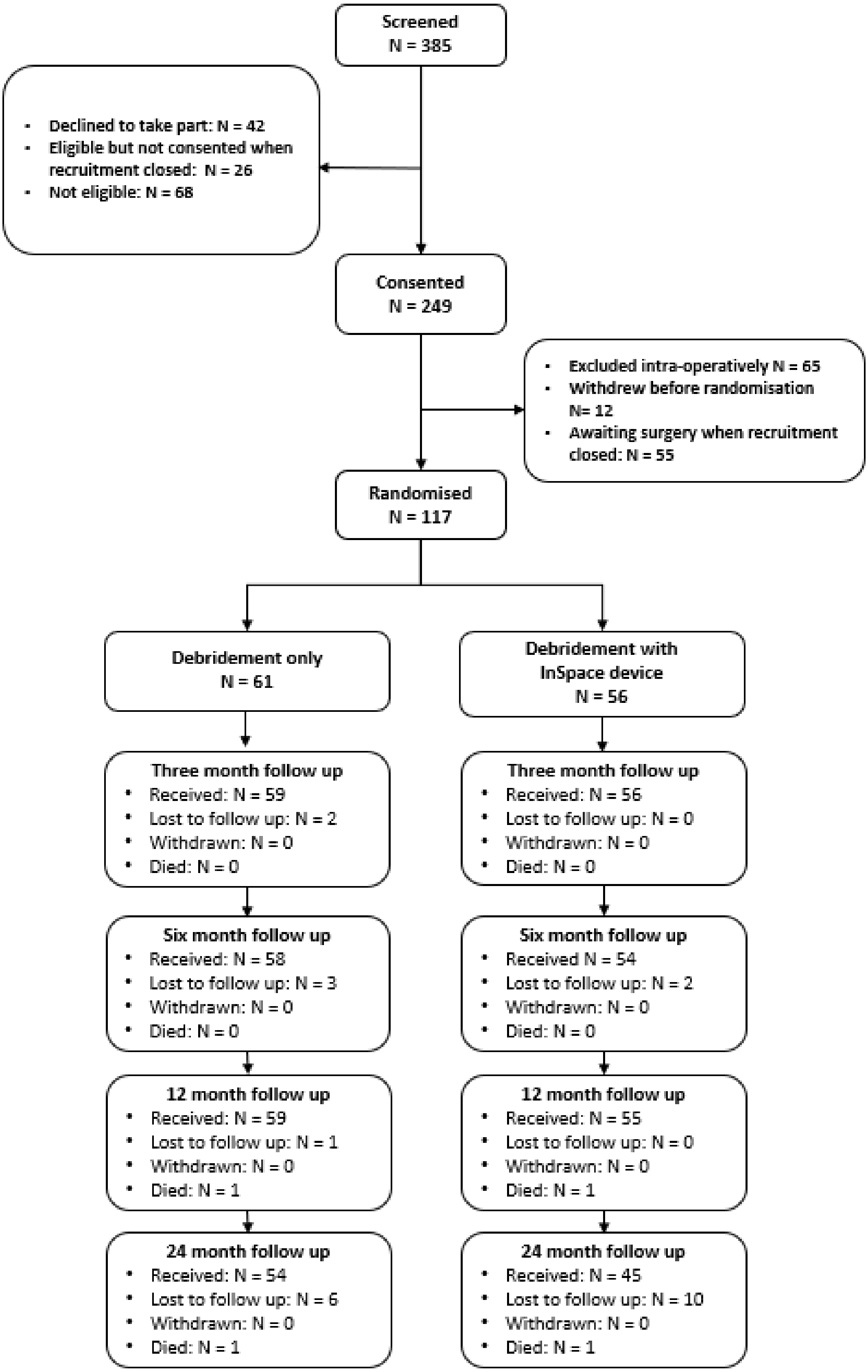

Table A1: All trial safety events (Complications and SAEs reported from 0-24 months)

**Complications**		**Debridement only (n=61)**	**Debridement with device (n=56)**	**Total (n=117)**
Participant experienced any complication (n, % of total participants in group)		13 (23)	13 (23)	26 (23)
Complications per participant (n)	1	10	10	20
	2 or more	3	3	4
Total complications (n)		16	20	36
Exacerbation/persistence of shoulder pain or restrictive range of motion (n, % of total complications)		6 (38)	6 (30)	12 (33)
Injection into the shoulder region (n, % of total complications)		1 (6)	3 (15)	4 (11)
Adhesive capsulitis (n, % of total complications)		0 (0)	2 (10)	2 (6)
Persistent muscle soreness or muscle injury (n, % of total complications)		0 (0)	1 (5)	1 (3)
Other (n, % of total complications)		5 (31)	4 (20)	9 (25)
Further surgery* (reverse shoulder Arthroplasty (n, % of total complications)		1 (6)	1 (5)	2 (6)
Persistent significant disability/incapacity*† (n, % of total complications)		3 (19)	2 (10)	5 (14)
Humerus fracture*† (n, % of total complications)		0 (0)	1 (5)	1 (3)

*Includes protocol defined SAEs. †Includes SAEs unrelated to study intervention

Figure A2 Distribution of Oxford Shoulder Scores at the 12 Month follow up

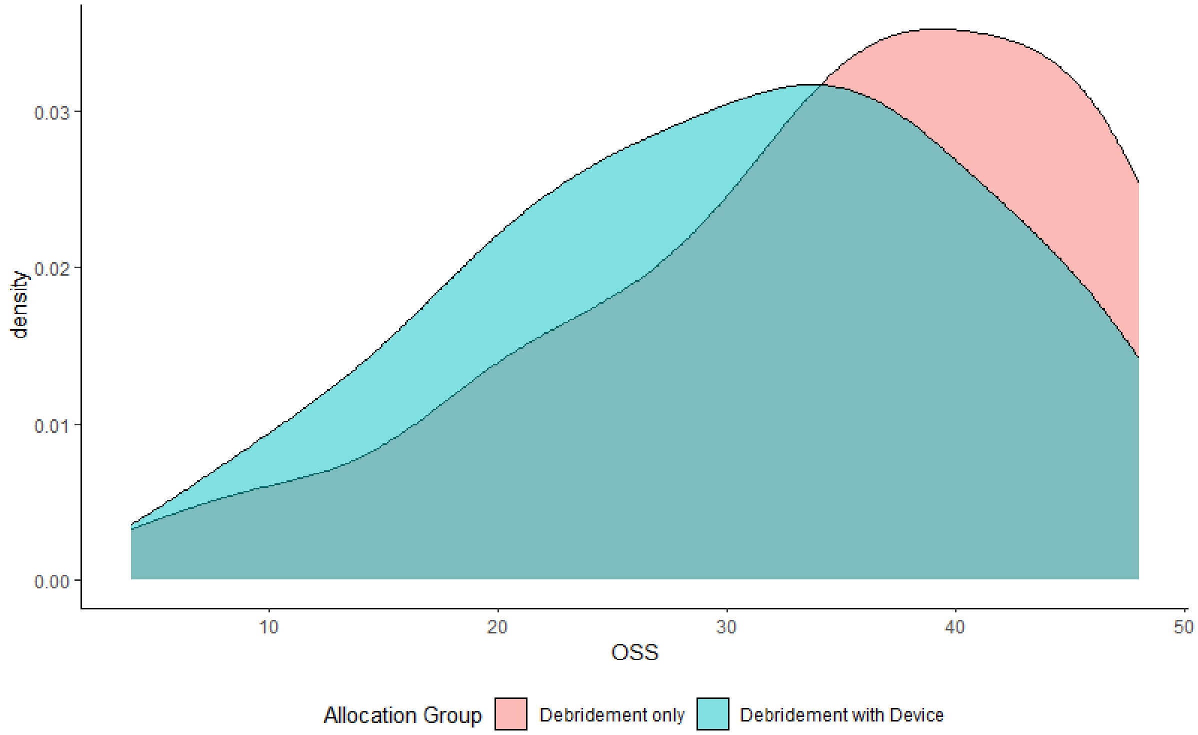

Figure A3 Distribution of Oxford Shoulder Scores at the 24 Month follow up

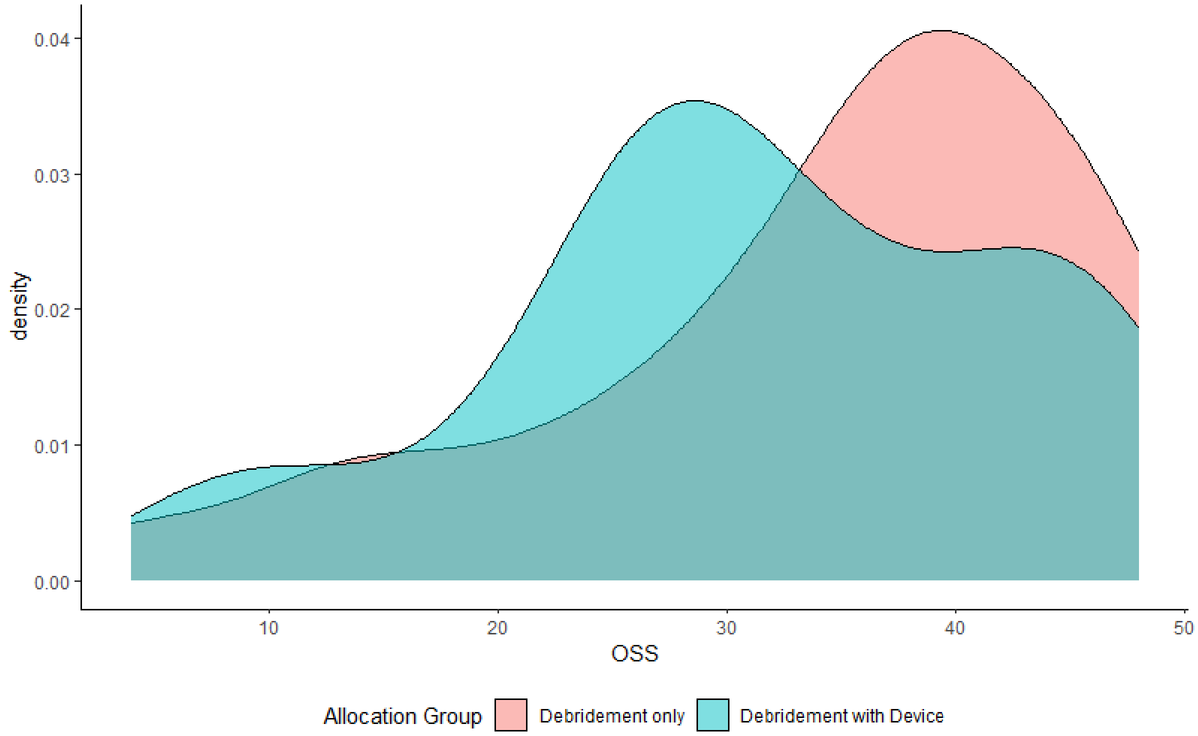

Table A2 Subgroup analyses: Interaction effects at 24 months

Fixed effect variables		Coefficient	95% CI	p-value
Intervention Group	Arthroscopy	0	-	0.94
	Arthroscopy with Device	-0.2	(-5.7, 5.3)	
Baseline	OSS score	0.6	(0.3,0.8)	p<0.001
Sex	Male	0	-	0.434
	Female	2.2	(0.4, 7.9)	
Tear Size	Large	0	-	0.971
	Medium or Small	-0.2	(-8.9, 8.6)	
Age group	Under 70	0	-	0.906
	70 and over	0.3	(-4.0, 4.5)	
Interaction Term	Arthroscopy with Device: Females	-7.8	(-15.9, 0.4)	0.06
